# Comparative Genomic Characterization of the Highly Persistent and Potentially Virulent *Cronobacter sakazakii* ST83, CC65 Strain H322 and Other ST83 Strains

**DOI:** 10.3389/fmicb.2017.01136

**Published:** 2017-06-26

**Authors:** Hannah R. Chase, Gopal R. Gopinath, Athmanya K. Eshwar, Andrea Stoller, Claudia Fricker-Feer, Jayanthi Gangiredla, Isha R. Patel, Hediye N. Cinar, HyeJin Jeong, ChaeYoon Lee, Flavia Negrete, Samantha Finkelstein, Roger Stephan, Ben D. Tall, Angelika Lehner

**Affiliations:** ^1^Center for Food Safety and Applied Nutrition, United States Food and Drug Administration, LaurelMD, United States; ^2^Institute for Food Safety and Hygiene, University of ZurichZurich, Switzerland; ^3^Quality Assurance and Food Safety Department, Hochdorf Swiss Nutrition LtdHochdorf, Switzerland

**Keywords:** *Cronobacter*, persister, environmental microarray, whole genome sequencing

## Abstract

*Cronobacter* (*C.*) *sakazakii* is an opportunistic pathogen and has been associated with serious infections with high mortality rates predominantly in pre-term, low-birth weight and/or immune compromised neonates and infants. Infections have been epidemiologically linked to consumption of intrinsically and extrinsically contaminated lots of reconstituted powdered infant formula (PIF), thus contamination of such products is a challenging task for the PIF producing industry. We present the draft genome of *C. sakazakii* H322, a highly persistent sequence type (ST) 83, clonal complex (CC) 65, serotype O:7 strain obtained from a batch of non-released contaminated PIF product. The presence of this strain in the production environment was traced back more than 4 years. Whole genome sequencing (WGS) of this strain together with four more ST83 strains (PIF production environment-associated) confirmed a high degree of sequence homology among four of the five strains. Phylogenetic analysis using microarray (MA) and WGS data showed that the ST83 strains were highly phylogenetically related and MA showed that between 5 and 38 genes differed from one another in these strains. All strains possessed the pESA3-like virulence plasmid and one strain possessed a pESA2-like plasmid. In addition, a pCS1-like plasmid was also found. In order to assess the potential *in vivo* pathogenicity of the ST83 strains, each strain was subjected to infection studies using the recently developed zebrafish embryo model. Our results showed a high (90–100%) zebrafish mortality rate for all of these strains, suggesting a high risk for infections and illness in neonates potentially exposed to PIF contaminated with ST83 *C. sakazakii* strains. In summary, virulent ST83, CC65, serotype CsakO:7 strains, though rarely found intrinsically in PIF, can persist within a PIF manufacturing facility for years and potentially pose significant quality assurance challenges to the PIF manufacturing industry.

## Introduction

*Cronobacter* is a genus of Gram-negative bacteria of the family *Enterobacteriaceae*. These bacteria have been associated with cases of illnesses in neonates for which fatality rates ranging between 40 and 80% have been reported (Tall et al., 2014). Clinical manifestations of infection include necrotizing enterocolitis, septicemia and neonatal meningitis. Infections in infants have been epidemiologically linked to consumption of contaminated batches of reconstituted powdered infant formula (PIF) (Hunter et al., 2008).

*Cronobacter* is capable of surviving under extreme desiccation (high osmotic stress) conditions and it is assumed that this characteristic contributes to its survival in PIF (and dried milk) factories, dried food products, and dry environments (Osaili and Forsythe, 2009; Osaili et al., 2009). Currently there are seven species described: *C. sakazakii*, *C. malonaticus, C. turicensis, C. universalis, C. condimenti, C. muytjensii*, and *C. dublinensis* all of which, except *C. condimenti* have been noted to cause infections in humans (Iversen et al., 2008; Joseph et al., 2012a). Of these species, *C. sakazakii* has been the most commonly isolated species from ill patients as well as from PIF products and production environments (Jolley and Maiden, 2010; Joseph et al., 2012b).

In April 2016 during routine testing of PIF packed products that were ready for distribution, a *C. sakazakii* isolate, H322, was recovered. A follow-up epidemiological investigation of this isolate and other isolates obtained from the facility’s production environment using macro restriction typing (Pulsed Field Gel Electrophoresis, PFGE) identified two other isolates which possessed indistinguishable PFGE patterns with H322, the oldest isolate dating back to 2012. This result suggested that these isolates were phylogenetically related and that the strain may have been persisting within the facility for more than 4 years (Stoller et al., 2016). Further characterization revealed that the strains possessed the serotype CsakO:7 LPS and sequence type (ST) ST83/CC65. In order to obtain further insights into the genes and features possibly responsible for the long-term persistence of these strains, whole genome sequencing (WGS), as well as microarray analysis, was applied to these strains as well as to one more ST83 isolate with a pulsotype distinct from this persistent clone. In addition, since no data on the putative pathogenicity of ST83 strains are available to date, we performed infection experiments in zebrafish embryos in order to assess information on the potential *in vivo* pathogenicity of the strains (Fehr et al., 2015; Eshwar et al., 2016).

## Materials and Methods

### Bacterial Strains

The five ST83 strains investigated in this study were isolated from PIF product (H322) and production environment (A31, H2399, H1191, H2397) in Switzerland between 2011 and 2016 during the routine hygiene monitoring performed by the PIF production company. The metadata associated with these strains is shown in **Table 1**. Putative *Cronobacter* strains were isolated according to the ISO/TS22964:2006 method. Isolates were identified using the genus- (alpha glucosidase) and species- (*rpoB*) specific PCR assays described by Lehner et al. (2006, 2012) and Stoop et al. (2009). Strains *C. sakazakii* ATCC 29544^T^ (C. sakO:1, ST8, clinical, child’s throat (United States), and NM1242 (C. sakO:4, ST4, clinical, brain exudate, United States) together with the non-pathogenic *E. coli* strain Xl1blue were included in zebrafish embryo infection studies (Eshwar et al., 2016).

**Table 1:** Details of *C. sakazakii* ST83 strains sequenced in this study.

Strain ID	Source	Date of isolation	O-type	ST/CC
H322	PIF product	21.04.2016	O:7	83/65
A31	PIF environment, floor drain	14.06.2012	O:7	83/65
H1191	PIF environment, vacuum cleaner	06.02.2012	O:7	83/65
H2399	PIF environment, vacuum cleaner	07.11.2011	O:7	83/65
H2397	PIF environment, vacuum cleaner	07.11.2011	O:7	83/65


### Preparation of Genomic DNA for PCR

Strains were grown in LB (Thermo Fisher Scientific AG, Zürich) at 37°C overnight and genomic DNA was isolated from 2 ml of culture using the Qiagen Blood and Tissue Kit according to the manufacturer’s instructions. 10–20 ng DNA was then used as the template in 50 μl PCR reactions.

### Serotyping PCR

O-antigen serotypes were determined by applying the scheme proposed by Sun et al. (2011).

### Multi-Locus Sequence Typing (MLST)

Multi-locus sequence typing was performed following the protocol described by Joseph et al. (2012b) or by submitting genome sequences using the *Cronobacter* MLST website^1^ (Jolley and Maiden, 2010). Sequencing of PCR amplicons was outsourced (Microsynth, Balgach, Switzerland).

### Genomic DNA Isolation for WGS and Microarray Analyses

All strains were grown overnight in a shaker incubator (160 rpm) at 37°C in 5 ml of Trypticase soy broth (BBL, Becton Dickinson, Franklin Lakes, NJ, United States) supplemented with 1% NaCl (final conc.). Isolation of genomic DNA was performed on 2 ml cultures using the robotic QIACube workstation and the automated Qiagen DNeasy technology (Qiagen, Inc.) following the manufacturer’s recommendations. Characteristically, 5–50 μg of purified genomic DNA was obtained in a final elution volume of 200 μl and used for plasmid typing and WGS analyses. For microarray analysis, the purified DNA was further concentrated using an Amicron Ultracel-30 membrane filter (30,000 molecular weight cut-off, 0.5 ml Millipore Corp.; Billerica, MA, United States) to a final volume of 10–25 μl as described by Tall et al. (2015).

### Microarray Analysis

The microarray used in this study is an Affymetrix MyGeneChip Custom Array (Affymetrix design number: FDACRONOa520845F) which utilizes the whole genome sequences of 15 *Cronobacter* strains, as well as 18 plasmids. These 15 strains encompassed all proposed species of *Cronobacter*. A ≥ 97% identity threshold level between gene homologs to positively predict allelic coverage as described by Tall et al. (2015) was used to design the array. Each gene is represented on the array by 22 unique 25-mer oligonucleotide probes, as described by Jackson et al. (2011) and Tall et al. (2015). Genomic DNA was hybridized, washed in the Affymetrix FS-450 fluidics station, and evaluated on the Affymetrix GeneChip^®^ Scanner 3000 (AGCC software) as described by Jackson et al. (2011) and as modified by Tall et al. (2015). All reagents for hybridizing, staining and washing were made in conjunction with the Affymetrix GeneChip^®^ Expression Analysis Technical Manual (Affymetrix, 2014). For each genetic locus represented on the microarray, probe set intensities were assessed using the Robust MultiArray Averaging (RMA) function in the Affymetrix package of R-Bioconductor as described by Bolstad et al. (2003). RMA summarization, normalization, and polishing was done on the data received and final probe set values were determined as explained by Jackson et al. (2011) and as modified by Tall et al. (2015). Gene differences were determined and phylogenetic trees were created using the SplitsTree 4 neighbor net joining method, and scatter plots were used for verification of the RMA-summarized probe set intensities as described by Jackson et al. (2011) and as modified by Tall et al. (2015).

### WGS Assembly and Comparative Genomic Analysis

Whole genome sequencing was carried out using the MiSeq platform (Illumina, San Diego, CA, United States), and a Nextera XT library kit. Trimmed Fastq data sets were *de novo* assembled through the CLC Genomics Workbench software version 7.0 (CLC bio, Aarhus, Denmark). WGS assemblies in FASTA format were submitted to NCBI under *Cronobacter* GenomeTrakr project as described by Grim et al. (2015) and Chase et al. (2016). Genome annotation was conducted through the RAST annotation server^2^ (Aziz et al., 2008; Overbeek et al., 2014). Genomes were submitted to NCBI under the *Cronobacter* GenomeTrakr Project: FDA-CFSAN bioproject number PRJNA258403 and accession numbers are given in **Table 2**. A local BLAST database was built to query and use with in-house perl scripts (will provide to users upon request). Homologs of conserved genes in these *Cronobacter* isolates were first identified using 4255 known CDS present in the NCBI GenBank annotation of *C. sakazakii* BAA-894 (GCA_000017665.1). Multiple sequence alignments to detect single nucleotide polymorphisms (SNPs) and Neighbor-Joining (NJ) algorithm based cladistic analysis of the resulting SNP matrix was carried out using MEGA7 suite (Kumar et al., 2016). To create a pseudomolecule of the pCS1-like native plasmid in H322, the WGS assembly was first mapped to pCS1 sequence (CP012254). The mapping of contigs was ordered in sequence and combined together to form an ungapped scaffold by manual curation. The resulting pseudomolecule represented the pCS1-like (pH322) sequences found in H322. Progressive Mauve implementation using Geneious 9 suite (Kearse et al., 2012) was used for global alignment.

**Table 2:** Overview of whole genomic characteristics of the *C. sakazakii* ST83 strains used in this study.

Strain ID	Genome size (mbp)	No. of contigs	%GC content	Number of coding sequences	NCBI Biosample ID	NCBI Accession No.
H322	4.6	44	56.7	4,255	SAMN06124518	MRXI00000000
A31	4.5	45	56.8	4,195	SAMN06124505	MRXM00000000
H1191	4.5	45	56.8	4,177	SAMN06124503	MRXQ00000000
H2399	4.5	36	56.8	4,152	SAMN06124500	MRXT00000000
H2397	4.4	143	56.8	4,136	SAMN06124502	MRXV00000000


### Zebrafish Embryo Infection Model

Husbandry, breeding, bacterial inoculum preparation, and infection of the embryos was carried out by microinjection of approx. 50 CFU of bacteria into the yolk sac of 2 dpf embryos according to the procedure described in the study by Eshwar et al. (2016). Virulence was assessed by determination of lethality (30 embryos per experiment/strain) over 72 h post-infection. The following controls were included: infection with (apathogenic) *E. coli* Xl1blue, injections with Delbecco’s Phosphate Buffered Saline (DPBS), and non-injected embryos. The maximum age reached by the embryos during experimentation was 120 hpf (72 hpi) for which no license is required from the cantonal veterinary office since embryos had not yet reached free feeding stage.

## Results and Discussion

Strain H322 was isolated from a contaminated batch of non-released PIF in April 2016. Subsequent PFGE analysis of *C. sakazakii* isolates obtained during the regular monitoring program performed by the production facility over the last 5 years revealed two more isolates (A31, H1191) exhibiting indistinguishable PFGE profiles, the oldest one dating back to February 2012 (data not shown). These results suggest that these strains have been persisting in the production environment for more than 4 years. Further analysis identified these strains as being of serotype O:7 and MLST ST83, CC65. Details on the strains are given in **Table 1**.

Isolates of this ST have been identified before during surveillance studies, most notably in environmental samples from this production facility (Müller et al., 2013; Stoller et al., 2016), but were rarely reported from other studies investigating PIF and PIF environmental isolates. In order to obtain a deeper insight into the genomic organization and the possible basis of the high persistence of these strains we performed WGS and microarray analyses on these clonal as well as one more, unrelated ST83 (H2399) strain. An overview of the WGS sequence data for the four strains is given in **Table 2**. Genome sizes ranged from 4.46 to 4.55 mbp, and their % G+C was approximately 56.8. The number of coding DNA sequences ranged from 4,136 to 4,255.

**FIGURE 1:**
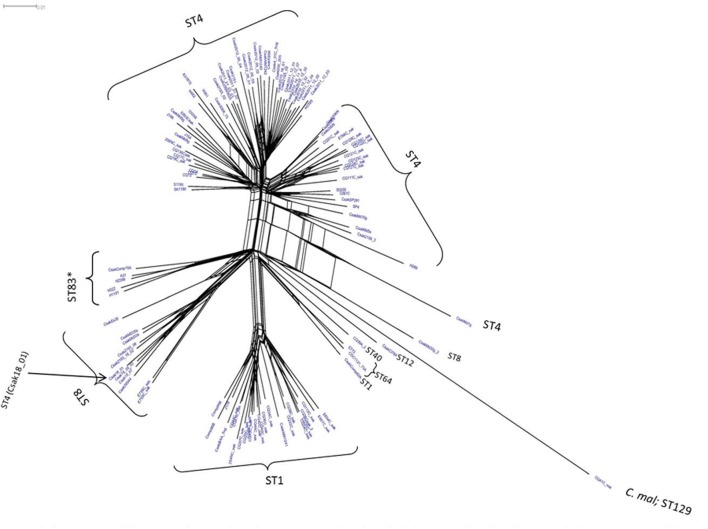
Phylogenetic Neighbor Net (SplitsTree4) analysis of 114 *C. sakazakii* and phylogenetically related strains, which were generated from the gene-difference matrix (Supplementary Tables S1, S2) and then overlaid with sequence types (ST). The microarray experimental protocol as described by Jackson et al. (2011) and as modified by Tall et al. (2015) were used for the interrogation of the strains for the analysis. The phylogenetic tree illustrates that the *Cronobacter* microarray could clearly separate the strains according to species and respective ST. The ST83 strains (^∗^) clustered together along with a single ST83 strain which was obtained from an United States diary powder manufacturing facility. Bar marker represents 0.01 gene differences.

**FIGURE 2:**
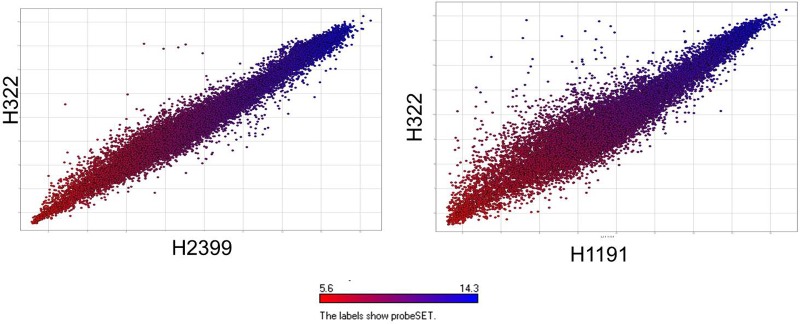
Comparative microarray probe set summarization of two Swiss ST83 strains H2399 and H1191 compared to strain H322 using scatter plots showing RMA summarized probe set intensities from each strain’s data points which are color-coded based on their intensities in H322. The *y*-axis show the RMA summarized probe set intensities from H322 (blue) and the *x*-axis shows the probe set intensities from H2399 and H1191 (red), respectively.

Microarray analysis of strains H322, A31, H1191 and H2399 compared to 109 other *C. sakazakii* strains and one *C. malonaticus* strain is shown in **Figure 1** (with supporting information given in Supplementary Tables S1, S2). The phylogenetic tree was first developed using the over 21,000 genes captured on the microarray and then MLST data was overlaid onto the strain clades. The majority of strains grouped according to STs. The Swiss ST83 strains clustered together with ST83 strain, CsakComp15A, which was obtained from a dairy powder manufacturing facility located in the United States. Noted exceptions were ST4 *C. sakazakii* strains 18_01 and Md1g which clustered with ST8 strains and as a singleton, ST8 strain Md33g clustered as a singleton, and ST1 strain Comp62A which clustered with a ST64 cluster, respectively. These results supports the hypothesis that MA using the over 21,000 *Cronobacter* genes captured on the microarray are more resolving than the seven-allele MLST scheme. Gene differences among the strains are shown in Supplementary Table S1 and demonstrate that the number of gene differences among strains A31, H3299, H322, and H1191 was between 5 and 38 genes. Metadata for the strains used in the microarray analysis is shown in Supplementary Table S2. RMA microarray probe summarization, visualized in scatter plots, yields a much lower variance in probe sets where intensities are less than 8 (log2) (Jackson et al., 2011). By decreasing this variance a more accurate determination of gene differences between strains is achieved. A summary of representative gene differences for strains H2399 and H1191 compared to strain H322 are shown in **Figure 2**.

**FIGURE 3:**
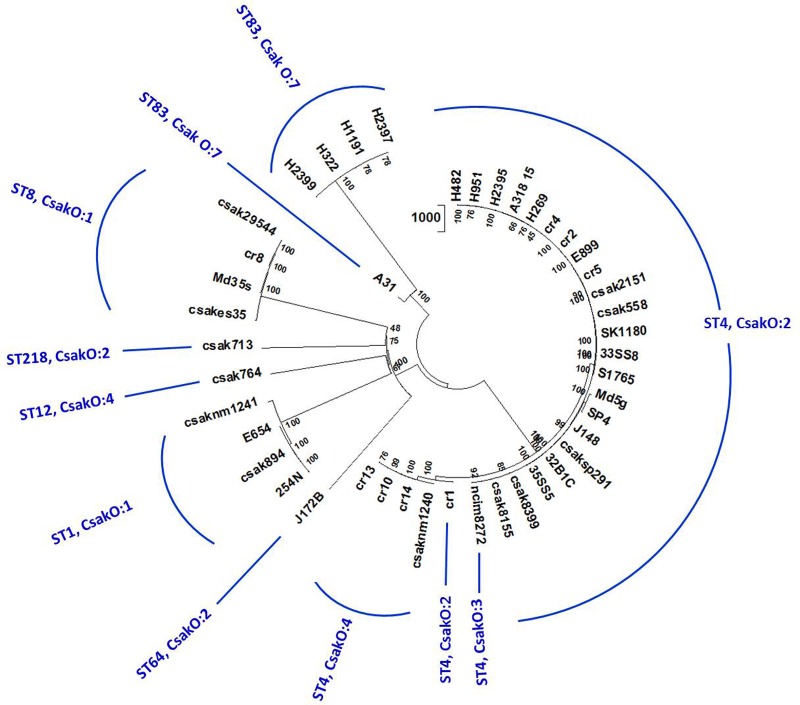
Phylogenetic analysis of *C. sakazakii* strains of different ST: Allelic variations in conserved genes spanning the whole genome were used for this analysis. Alleles present in homologs of 1000 *C. sakazakii* strain BAA-894 chromosomal loci (reference gene set for this analysis) in 44 genomes were detected by BLAST-based workflow (Tall et al., 2015). SNPs in 37,711 positions in these genomes were concatenated and the cladogram was built using Neighbor-Joining algorithm available on MEGA7 phylogenetic suite (Kumar et al., 2016). The bootstrap consensus (values located on the nodes) in the tree are inferred from 500 replicates. 44 genomes separated into eight STs and multiple clusters reflecting microarray-based clusters. Five ST83 isolates group together with A31 showing some differences in the cluster confirming other results.

Homologs of 4,024 (of the 4255 genes previously identified) *C. sakazakii* BAA-894 genes conserved in ST83 and other strains used in the study were identified by local BLAST analysis as described earlier. SNPs associated with 37,711 positions across 44 genomes in 1,000 randomly chosen, conserved loci were detected using a local BLAST database and aligned to create an un-gapped dataset. A high-confidence unrooted phylogenetic tree based on the NJ algorithm in MEGA7 program is shown in **Figure 3** (supporting information is given in Supplementary Table S3) and revealed that the ST83 strains formed their own distinct cluster. Similar to the microarray-based cladistics analysis, small but distinct differences among the lineages of *C. sakazakii* strains from food, clinical and environmental samples are captured in this conserved-loci-based SNPs analysis. As expected, this high throughput multi loci SNP analysis was able to group isolates belonging to same ST into distinct clusters.

Whole genome sequencing analysis also showed that strain H322 harbors a pESA3/pCTU1-like virulence plasmid, which was previously found by whole-genome sequencing in strains *C. sakazakii* BAA-894 (NC_00978) and *C. turicensis* z3032 (NC_01328). The pESA3/pCTU1-like plasmid harbored by H322 (107,409 bp) shares 97% identity over a 75% query coverage when aligned with the pESA3 harbored by *C. sakazakii* BAA-894, and 90% identity of a 58% query coverage when aligned with *C. turicensis* z3032’s pCTU1 (Franco et al., 2011). PCR analysis targeting the prevalence and distribution of plasmid targets is shown in **Table 3** which supports these genomic findings (Franco et al., 2011). Additionally, nucleotide sequences encoding plasmid partitioning proteins ParA and ParB, as well as the IncFIB plasmid conjugative transfer surface exclusion protein TraT, were found in H322 (Kucerova et al., 2010; Franco et al., 2011). Interestingly, the other four strains additionally possessed the *repA* gene, signifying that these strains also harbor a pESA3-like plasmid. Typical pESA3 plasmid genes such as *eitA*, *iucC*, *cpa* and some regions of the type six secretion system were also possessed by these strains. Additionally, strain H1191 was found to possess the *repA* gene encoded on the pESA2-like plasmid.

**Table 3:** Plasmid typing results for the *C. sakazakii* serotype ST 83 strains analyzed in this study.

	PCR reaction results^∗^														
	
Isolate Name	pESA3	eit	iuc	cpa	Δcpa	ΔT6SS	T6SS IntL	vgrG	T6SSR end	T6SS IntR	Δfha	fha	dfha	pESA2	pCTU3
A31	(+)	(+)	(+)	(+)	(+)	(-)	(+)	(-)	(-)	(+)	(+)	(-)	(-)	(-)	(-)
H2399	(+)	(+)	(+)	(+)	(+)	(-)	(+)	(-)	(-)	(+)	(+)	(-)	(-)	(-)	(-)
H2397	(+)	(+)	(+)	(+)	(+)	(-)	(+)	(-)	(-)	(+)	(+)	(-)	(-)	(-)	(-)
H1191	(+)	(+)	(+)	(+)	(+)	(-)	(+)	(-)	(-)	(+)	(+)	(-)	(-)	(+)	(-)
H322	(+)	(+)	(+)	(+)	ND	ND	(+)	(-)	(-)	(-)	(+)	(-)	ND	(-)	(-)


^∗^Results are based on description of PCR amplification of reactions using primers and parameters described by Franco et al., (2011). For example (+) refers to an amplification of targeted gene; (-) refers to a negative amplification result, and ND refers to not determined. ND results for Δ*cpa* and *fha* are due to positive PCR findings of the presence of *cpa* and the conserved flanking regions of *fha* in these strains. Negative PCR results for these targets are expected.

**FIGURE 4:**
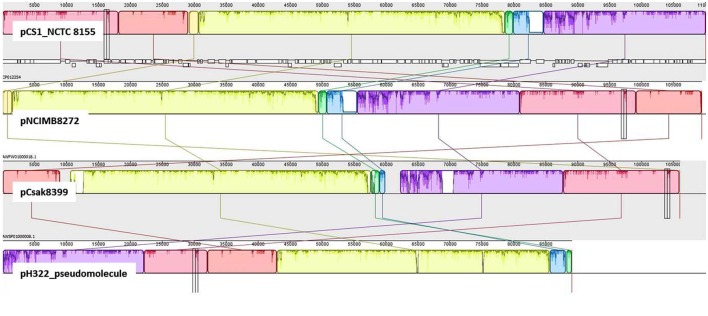
Comparative genomic analysis to identify pCS1-like sequences: Mauve global alignment of the pCS1-like sequences in H322: (track 4) with *C. sakazakii* NCTC 8155 pCS1_NCTC 8155 (track 1), *C. sakazakii* pNCIM8272 (track2) and *C. sakazakii* 8399 pCsak8399 (track 3). H322 contigs mapping to pCS1 plasmid (CP012254) were used to create an artificial molecule to represent pH322 (track 4; pH322_pseudomolecule). This artificial scaffold was compared with pCS1 and pCS1-like plasmid contigs from strains NCIMB8272 (track2) and Csak8399 (track3). In addition to pESA3-like plasmid, isolates such as strain H322 reported in this work contain a large plasmid highly comparable to pCS1 plasmid.

In addition to pESA3, the presence of a large plasmid in these ST83 genomes was also found. Detailed analysis revealed that this plasmid was significantly orthologous to the 110 kb plasmid pCS1 reported from *C. sakazakii* NCTC 8155 (Moine et al., 2016). Using BLAST analysis and manual curation, the WGS contigs representing about 80% of the pCS1 sequence (CP012254) in *C. sakazakii* strain H322, were used to create a gapped scaffold of 89 kbp (pH322_pseudomolecule). In **Figure 4**, the pH322 pseudomolecule was aligned with highly conserved pCS1-like contigs from NCIM8272 (contig 22-AWFW01000018) and Csak8399 (contig11-AWSP01000008) using an implementation of ProgressiveMauve algorithm in Geneious suite 9.1 (Darling et al., 2010; Kearse et al., 2012). The pCS1-like sequences in H322 (track 4) is shown to align with *C. sakazakii* NCTC 8155 pCS1_NCTC 8155 (track 1), *C. sakazakii* pNCIM8272 (track2) and *C. sakazakii* 8399 pCsak8399 (track 3). This analysis showed that large sequence regions within the plasmids typified in each track shared significant conservation to the reference (pCS1 plasmid CP012254). None of these sequences showed any similarity to pESA3 and/or pCTU1 virulence plasmids. In addition to the ST83 isolates from this study and the two other strains from this study as illustrated in **Figure 3**, pCS1-like sequences are present in *C. sakazakii* 558 and *C. malonaticus* 685 genomes (Supplementary Table S2) downloaded from NCBI. It is evident from this comparative analysis that the pCS1-like plasmid found in ST83 strain H322 (and others): (i) represents a new kind of high-molecular weight plasmid among *C. sakazakii* strains orthologous to the recently reported pCS1 (Moine et al., 2016); (ii) belongs to an unknown incompatibility class but contains a homolog of *parB* found in *S. enterica* serovar Typhimurium plasmid pSTM_Phi (Octavia et al., 2015); and (iii) may exist in *C. sakazakii* lineages in addition to pESA3-like plasmids.

Interestingly, the genome of H322 harbors 43 gene sequences related to efflux-pump activity, such as those from multidrug resistance gene families and superfamilies such as RND, AcrR (the negatively acting repressor part of the *acrRAB* operon), MFS, PET, arsenic, DMT, and MATE. Furthermore, H322 has eleven alleles related to stress response, including: *degQ* and *degS*, and universal stress protein genes A, B, C, E, and G, as well as 25 alleles related to polysaccharide metabolism, transportation, exportation and biosynthesis. For comparison, additional genes found in each of the strains by RAST analysis are shown in Supplementary Table S4.

**FIGURE 5:**
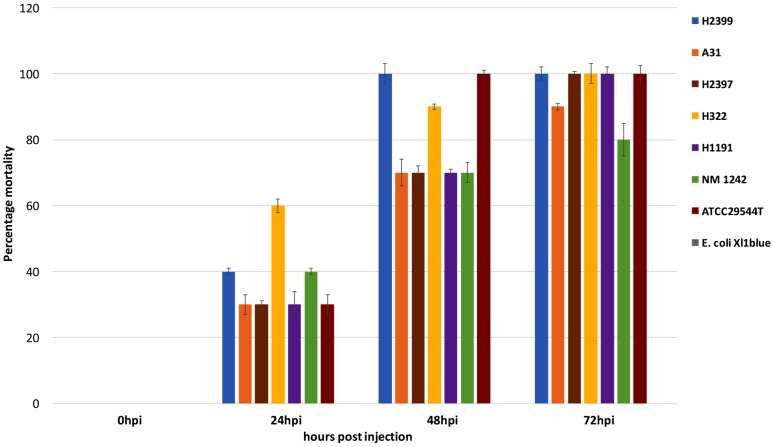
Results of the zebrafish embryo infection experiments with five *C. sakazakii* ST83 strains, the two clinical strains ATCC29544^T^ and NM 1242 as well as *E. coli* Xl1 blue.

As of now, there are only two ST83 clinical isolates known which includes one from Israel (1998) and the other from China (2014) that have been submitted to the *Cronobacter* pubMLST site^3^ (Jolley and Maiden, 2010). Studies on the potential virulence of *C. sakazakii* strains have been limited in the past due to the lack of suitable animal models by which strains and mutants may be analyzed in a high-throughput manner. In 2015 the zebrafish embryo model was adapted for infection studies in *Cronobacter* spp. which was intended to meet this requirement (Fehr et al., 2015; Eshwar et al., 2016). The use of this model made it possible to analyze strains of non-clinical origin for their virulence potential *in vivo*. In the current study the ST83 strains were subjected to infection studies and the results revealed high (90–100% lethality within 3 days) virulence potential for these ST83 strains (**Figure 5**). As controls, two clinical *C. sakazakii* isolates (ATCC 29544^T^, NM1242) of other STs (ST8 and ST4, respectively) as well as *E. coli* Xl1 blue were included in these experiments. While the *C. sakazakii* type strain ATCC29544^T^ (isolated from a child’s throat) showed 100% lethality in the assay, the NM1242 (isolated from brain exudate) strain exhibited a slightly reduced lethality in zebrafish embryos. Infections with *E. coli* Xl1 blue (as well as all other controls) were asymptomatic, as expected. Although – to date – no illnesses have been linked to infections with ST83 strains, our results cannot rule out a potential risk for neonates exposed to PIF contaminated with *C. sakazakii* ST83 strains.

Infection studies with *C. sakazakii* of STs other than ST83, including *C. sakazakii* ST4 strains, which have been linked to neonatal infections and are frequently isolated from PIF and PIF environments, are currently ongoing. The availability of the H322 genome will enable its comparison with other genomes of *C. sakazakii* strains, providing more insights into genetic features encoding stress resistance, efflux pump activity, plasmid presence, and polysaccharide metabolism that are associated with this foodborne pathogen.

## Author Contributions

All authors (HRC, GG, AE, AS, CF-F, JG, IP, HNC, HJ, CL, FN, SF, RS, BT, and AL) contributed to the drafting of the manuscript. HRC, GG, AE, JG, IP, HNC, HJ, CL, FN, SF, RS, and BT carried out the data acquisition, analysis and interpretation of data. AE performed the zebrafish infection experiments and AE and AL interpreted the results. HRC, GG, AE, JG, IP, HNC, HJ, CL, FN, SF, and BT performed the whole genome sequencing and microarray experiments and GG interpreted the results. All authors contributed to the study concept and design; and critical revision of the manuscript for important intellectual content.

## Conflict of Interest Statement

The authors declare that the research was conducted in the absence of any commercial or financial relationships that could be construed as a potential conflict of interest.

## Footnotes

1 http://pubmlst.org/cronobacter/

2 http://www.nmpdr.org/FIG/wiki/view.cgi/FIG/RapidAnnotationServer

3 http://pubmlst.org/cronobacter/
